# The Search for Consumers of Web-Based Raw DNA Interpretation Services: Using Social Media to Target Hard-to-Reach Populations

**DOI:** 10.2196/12980

**Published:** 2019-07-30

**Authors:** Tiernan J Cahill, Blake Wertz, Qiankun Zhong, Andrew Parlato, John Donegan, Rebecca Forman, Supriya Manot, Tianyi Wu, Yazhu Xu, James J Cummings, Tricia Norkunas Cunningham, Catharine Wang

**Affiliations:** 1 Divison of Emerging Media Boston University College of Communication Boston, MA United States; 2 Department of Community Health Sciences Boston University School of Public Health Boston, MA United States

**Keywords:** research subject recruitment, social media, survey methods, data collection methods, advertising as topic, algorithms

## Abstract

**Background:**

In recent years, there has been a proliferation of third-party Web-based services available to consumers to interpret raw DNA from direct-to-consumer genetic testing companies. Little is known about who uses these services and the downstream health implications. Identifying this hard-to-reach population of consumers for research raised questions about the most effective recruitment methods to undertake. Past studies have found that Web-based social media survey distribution can be cost-effective for targeting hard-to-reach populations, yet comparative efficacy information across platforms is limited.

**Objective:**

The aim of this study was to identify the most effective Web-based strategies to identify and recruit the target population of direct-to-consumer genetic testing users who also made use of third-party interpretation services to analyze their raw genetic data. Web-based survey recruitment methods varying by social media platform and advertising method were compared in terms of cost-effectiveness and demographics of survey respondents.

**Methods:**

A total of 5 Web-based survey distribution conditions were examined: 4 paid advertising services and 1 unpaid service. For the paid services, a 2x2 quasi-experimental design compared social media platforms (Facebook vs Twitter) and advertising tracking metrics (by click vs by conversion). The fifth unpaid comparison method consisted of study postings on the social media platform, Reddit, without any paid advertising. Links to identical Web-based versions of the study questionnaire were posted for 10 to 14 days for each of the distribution conditions, which allowed tracking the number of respondents that entered and completed the questionnaire by distribution condition.

**Results:**

In total, 438 individuals were recruited to the study through all conditions. A nearly equivalent number of participants were recruited from paid campaigns on Facebook (n=159) and Twitter (n=167), with a smaller sample recruited on Reddit (n=112). Significantly more participants were recruited through conversion-tracking (n=222) than through click-tracking campaigns (n=104; *Z*=6.5, *P*<.001). Response rates were found to be partially driven by organic sharing of recruitment materials among social media users. Conversion tracking was more cost-effective than click tracking across paid social media platforms. Significant differences in terms of gender and age distributions were noted between the platforms and between the tracking metrics.

**Conclusions:**

Web-based recruitment methods were effective at recruiting participants from a hard-to-reach population in a short time frame. There were significant differences in the effectiveness of various paid advertising techniques. Recruitment through Web-based communities also appeared to perform adequately, yet it may be limited by the number of users accessible in open community groups. Future research should evaluate the impact of organic sharing of recruitment materials because this appeared to play a substantial role in the observed effectiveness of different methods.

## Introduction

To date, there have been a number of inquiries into using social media for research recruitment and there has been little consensus in terms of results. A systematic review of 30 existing studies on social media recruitment found mixed evidence with regard to the efficacy of survey recruitment on social media but did find that such methods were consistently found to be effective when specifically targeting hard-to-reach populations—those that are difficult to find or involve in research and public health programs because of their geographical location or socioeconomic situation [1,2]. However, the review also suggested that this methodology has not been studied often enough to generate firm conclusions as to its efficacy, arguing that further research, particularly research examining the cost-efficacy of different recruitment techniques and demographic differences in the resulting samples, is necessary [1]. This study partially fills this gap in the research by directly comparing multiple analogous advertisement recruitment methods on Twitter and Facebook along with the unpromoted posts on Reddit to recruit survey respondents from the same hard-to-reach population.

Social media has been defined, in a public health context, as websites that allow users to create profiles and use those profiles to connect and interact with other users [1]. Although there are dozens to hundreds of different forms of social media, at present, most of the documented social media recruitment efforts for population health research have used Facebook [3-9]. Facebook is a large social media platform with approximately 1.56 billion daily active users [10]. Studies have found success in reaching target audiences by sharing posts within Facebook communities, enlisting respondents in *snowball sampling* campaigns, and purchasing paid advertising on the platform targeting specific demographics. Although there have been fewer studies focusing on the platform, Twitter—a somewhat smaller platform with 126 million daily active users [11]—has also been used for recruitment purposes both through researchers tweeting and retweeting recruitment tweets [12,13] and advertisements [14].

There has been little research to compare the effectiveness of recruitment from across different social media platforms, although some studies have sought to use multiple platforms for recruitment without making direct statistical comparisons [15,16]. One study did compare 2 social media platforms (Twitter and Facebook) as well as another method (distributing quick response codes through mail) but did not use directly analogous recruitment methods across conditions or identify platform-level differences [17]. As a result, direct comparisons of relative effectiveness between the platforms themselves remain a challenge.

Survey research employing social media for participant recruitment has also yet to consider the multiple recruitment strategies available on a given platform. Popular platforms, such as Facebook, enable both *cost per click* advertisement sales that charge advertisers each time a user clicks on an advertisement and *cost per conversion* sales: Advertisers are billed on the basis of specific, predefined actions that follow from a user clicking through the advertisement, such as purchasing a product or completing a questionnaire. These tracking metrics may yield different results when it comes to reaching target audiences as well as achieving a cost-effective survey sample.

This study sought to better understand the differences in survey participant recruitment between social media platforms, as well as within-platform differences resulting from different tracking methods. Targeted survey participants were a hard-to-reach population of users of direct-to-consumer genetic testing (DTC-GT) services (eg, AncestryDNA and 23andMe) who had subsequently used third-party interpretation tools to analyze their raw genetic data. The goal of the study was to compare the cost-effectiveness as well as the demographic characteristics of the sample across different platforms and between different advertising tracking metrics.

To enable a more rigorous comparison between different social media platforms, this study conducted advertising campaigns on both Facebook and Twitter—a platform deemed to possess sufficient similarities to Facebook in terms of advertising affordances and presentation of content so that comparisons can be made. In addition, click-based and conversion-based tracking metrics were used on each platform. To allow for further comparisons across social media platforms, an additional condition contrasted unpaid posts to community message boards on Reddit with the advertising campaigns on Facebook and Twitter.

This study addressed the following questions regarding platform differences, cost-efficacy, and paid versus unpaid uses of social media in survey recruitment:

Q1: Among paid social media campaigns, which social media platform is most effective at generating survey responses from the hard-to-reach population of DTC-GT users who had also used third-party interpretation tools?

Q2: Among paid social media campaigns, which advertising tracking method is most effective at generating survey responses from the hard-to-reach population of DTC-GT users who had also used third-party interpretation tools?

Q3: Do surveys conducted via paid social media campaigns on Facebook and Twitter generate more survey responses from the hard-to-reach population of DTC-GT users who had also used third-party interpretation tools compared with surveys posted on open (unpaid) Web-based communities?

Q4: What demographic differences exist between survey respondents who are recruited using (1) different platforms and (2) different advertising tracking methods?

## Methods

This study compared the effectiveness and cost of different social media recruitment methodologies that comprised both paid and unpaid advertising structures across different platforms (Facebook, Twitter, and Reddit). Despite its large user base (1 billion active users), Instagram was not included because of the lack of a well-defined community of interest, which was the basis for targeting advertisements toward the relevant population. The target population for the survey was defined as US residents who had undergone genetic testing via direct-to-consumer (DTC) companies and who subsequently used third-party Web-based DNA interpretation services.

### Paid Recruitment Methods

**Figure 1:**
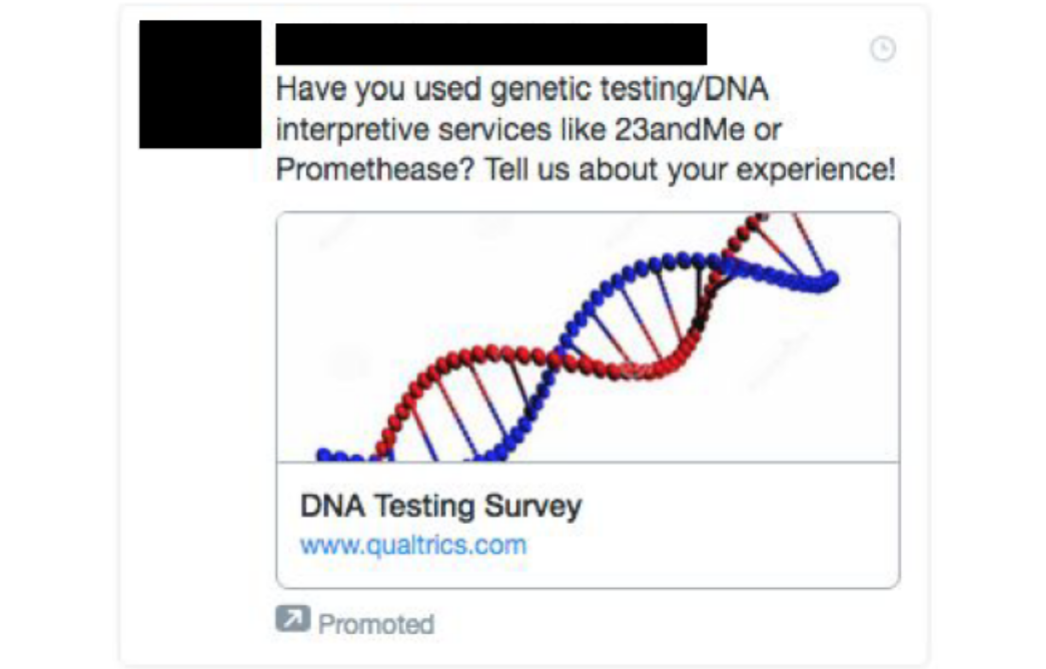
Example of recruitment materials on Twitter.

**Figure 2:**
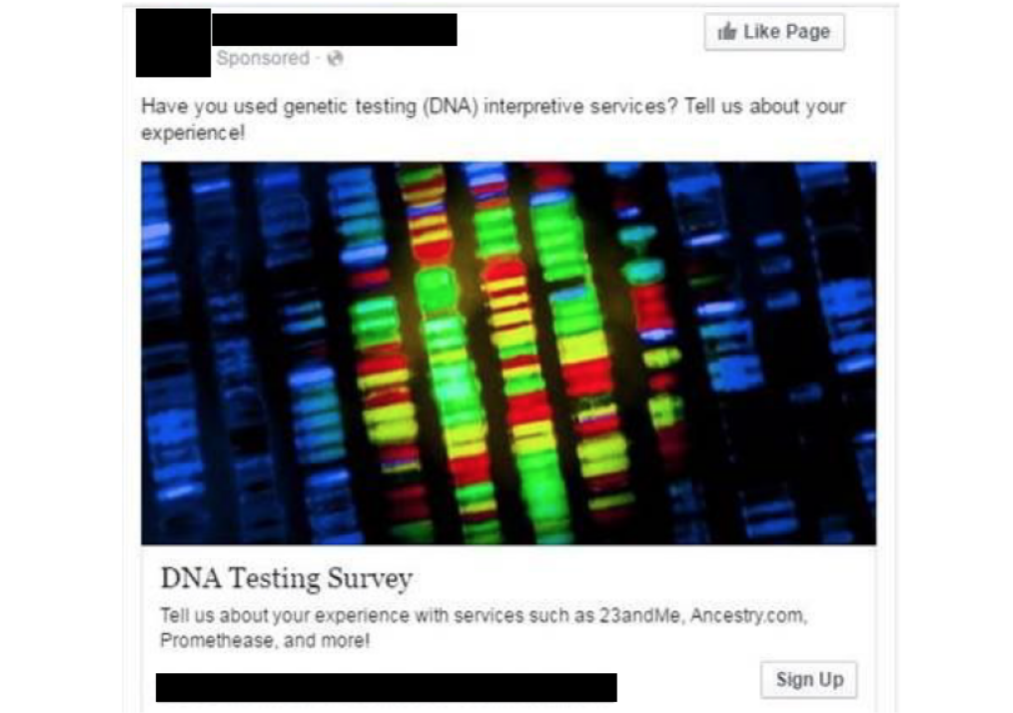
Example of recruitment materials on Facebook.

A 2x2 factorial design was used to test the comparative effectiveness and cost of different platforms and advertising tracking metrics for paid recruitment. Facebook and Twitter were selected as the platforms to be compared based on their large US resident user bases. Both platforms have proprietary content distribution networks that distribute paid advertising content to their users. Advertised content appeared as *promoted* status cards or tweets in the news feed of the targeted users (Figures 1 and 2), intermingled with user-generated content.

There are slight differences in the way each content distribution network allows for targeting of specific user demographics. An effort was made to mirror the approach taken to targeting users across both sites. On Facebook, the potential audience of the campaign was defined as users living in the United States with an interest in 23andMe, a major DTC-GT company. Facebook targets paid advertising campaigns by identifying *interests from information users have added to their Timeline, keywords associated with the Pages they like or apps they use, ads they have clicked on, and other similar sources* [18]. All users aged 18 years and older were included, resulting in a potential audience for the campaign of 740,000 Facebook users at the time of launch.

On Twitter, the potential audience of the campaign was defined as US-based Twitter users who were followers of @23andMe’s Twitter account, as well as users with interests similar to followers of @23andMe. According to Twitter, “[Follower targeting] works by displaying your Twitter Ads campaigns to people who follow specific usernames or are similar to the followers of those usernames” [19]. This resulted in a potential audience size for the campaign of between 178,000 and 267,000 Twitter users at the time of launch.

A total of US $1000 was budgeted for the paid campaigns, divided evenly between Facebook and Twitter. Automatic bidding was the default on both platforms and was used in all 4 conditions. This feature dynamically adjusts the cost of advertising based on availability and demand, as well as the bidding parameters set by other advertisers. There are minor differences in the way each advertising platform handles bidding for advertisements: Twitter requires advertisers to specify a daily budget and provides an optional total budget setting for automatic bidding, after which the campaign will end. The daily budget for each condition was set at US $25 per day with a total budget of US $250 per campaign. Facebook does not require a daily budget setting; however, the total budget for this campaign was also set at US $250. Each campaign was allowed to run until the total budget was exhausted: Twitter advertisements ran for 10 days each, whereas Facebook advertisements were displayed for 14 days each. Although both Facebook and Twitter provide advertisers some control over the time of the day when advertisements are displayed, it was not specified on either platform in this study.

On both social media platforms, 2 advertising campaigns were conducted using different payment structures corresponding to different tracking methods. Both platforms allow the advertiser to either pay for each click through to the advertiser’s landing page (cost per click) or to pay for each iteration of a defined conversion action after the user has clicked through to the landing page (cost per conversion). For the purposes of the study, a *conversion* was defined as the user reaching the end of the questionnaire.

Both advertising platforms claim to iteratively *optimize* the targeting of a given advertising campaign based on the tracking metric used. Thus, a campaign for which the advertiser is billed per click is purportedly targeted in such a way as to maximize the likelihood that a given user who is shown the content will click on it. Conversely, a campaign for which the advertiser is billed per conversion is purportedly targeted in such a way as to maximize the likelihood that a given user will complete the conversion action after having clicked through.

To track which of the users that were shown the recruitment material ended up completing the questionnaire and allowing feedback to the content distribution network for optimization purposes, a tracking pixel was used for the 2 campaigns in the conversion-based condition. A tracking pixel is a hidden image file embedded in a custom landing page, which users were automatically redirected to after having completed the questionnaire. Loading the image in a Web browser triggers a JavaScript function on the page, which logs the conversion with either Twitter or Facebook, depending on which version of the questionnaire was completed. In contrast, the 2 campaigns using the click-based condition only tracked how many users clicked the link to the questionnaire rather than any user interaction with the questionnaire. Each campaign used a separate, yet identical, Web-based questionnaire, enabling survey respondents to be categorized by the advertising campaign that recruited them.

### Unpaid Recruitment Method

A parallel recruitment campaign was conducted on Reddit, a social news and community discussion site, to assess the viability of recruitment through unpaid posts to relevant Web-based communities. Reddit was selected because of the presence of several relevant community groups (see the table provided in Multimedia Appendix 1) as well as the open structure of the site, which allows any user to post to any public group or subreddit, subject to community moderation. In total, 13 relevant subreddits were identified, although r/Health was not used because of community guidelines that prohibited the posting of content other than news. Identical posts were made on each of the remaining 12 subreddits seeking respondents for the survey (see the textbox provided in Multimedia Appendix 1).

### Statistical Analysis

#### Data Preparation

The dataset was screened for duplicate responses using the internet protocol (IP) address and demographic profile of respondents, where responses from the same IP address within a 24-hour period or responses from the same IP address with a matching demographic profile were flagged as duplicates. This resulted in 17 responses being removed from the subsequent analysis. No responses were found to have been duplicated more than once, suggesting that these were likely the result of user error rather than a systematic effort.

#### Recruitment Effectiveness

A chi-square test was conducted to determine the extent to which the proportion of observed frequencies among the 4 paid campaigns conformed to a discrete uniform distribution, which would suggest the absence of a measurable difference in recruitment effectiveness between conditions. Posthoc pairwise *Z* tests were performed between all campaigns with a Bonferroni correction for multiple comparisons.

#### Cost-Effectiveness

The recruitment budget for each condition was fixed at US $250, such that more cost-effective methods would yield a greater total number of responses over the study period. On the basis of this fixed budget, the cost-efficacy of each paid campaign was calculated in terms of the cost per survey response and cost per 1000 impressions. Each *impression* marks a time when the recruitment materials were displayed to a user, regardless of whether that user had seen the materials before or interacted with them in any way.

#### Survey Demographics

A total of 4 demographic variables were collected in the survey: age, gender, education, and race and ethnicity. Chi-square tests of homogeneity were performed to determine the statistical significance of differences in the distributions of gender and ethnicity. Participants who reported their gender as neither male nor female were excluded from the analysis of gender distributions because of the absence of reliable information on the expected proportion of nonbinary gender identifying individuals in the population. Age distributions were compared using a one-way analysis of variance. Posthoc pairwise comparisons were conducted with a Bonferroni correction, as appropriate. Kruskal-Wallis H tests were used to compare the education level of respondents. To maximize the response rate, demographic questions were not required to complete the survey. For demographic analyses only, participants who did not report the demographic characteristic of interest were excluded.

## Results

### Study Participants

Participant demographics are presented in Table 1. Notably, because demographic questions were optional in the questionnaire, a substantial portion of respondents who completed the rest of the questionnaire elected not to answer them.

The mean age of those who reported this (n=266) was 46 years at the time of the survey. Among respondents who reported their gender (n=298), the majority (204/298, 68.5%) were female. The median level among those who reported their level of education (n=296) was a 4-year college degree across all conditions. Among respondents who reported their race or ethnicity (n=294), the majority (238/294, 81.0%) were white.

**Table 1:** Participant demographics.

Demographic variables		Participants (N=438), n (%)	Participants (excluding missing), n (%)
**Age (years)**			**N=266**
	18-24	24 (5.5)	24 (9.0)
	25-44	101 (23.1)	101 (38.0)
	45-64	109 (24.9)	109 (41.0)
	65 and older	32 (7.3)	32 (12.0)
	Did not report	172 (39.3)	—^a^
**Gender**			**N=298**
	Female	204 (46.6)	204 (68.5)
	Male	93 (21.2)	93 (31.2)
	Other	1 (0.2)	1 (0.3)
	Did not report	140 (32.0)	—
**Education**			**N=296**
	Less than high school	3 (0.7)	3 (1.0)
	High school/GED^b^	11 (2.5)	11 (3.7)
	Some college	63 (14.4)	63 (21.3)
	2-year college degree	39 (8.9)	39 (13.2)
	4-year college degree	91 (20.8)	91 (30.7)
	Advanced degree (postgraduate)	89 (20.3)	89 (30.1)
	Did not report	142 (32.4)	—
**Race and Ethnicity**			**N=294**
	White/Caucasian	238 (54.3)	238 (81.0)
	African American	7 (1.6)	7 (2.4)
	Hispanic/Latino	10 (2.3)	10 (3.4)
	Asian	8 (1.8)	8 (2.7)
	Multiethnic	23 (5.3)	25 (7.8)
	Other	8 (1.8)	8 (2.7)
	Did not report	144 (32.9)	—

^a^Valid percentage excludes respondents who did not report for a given demographic variable.

^b^GED refers to those respondents who reported completing the General Education Development tests as their highest level of educational attainment.

### Recruitment Effectiveness

**Figure 3:**
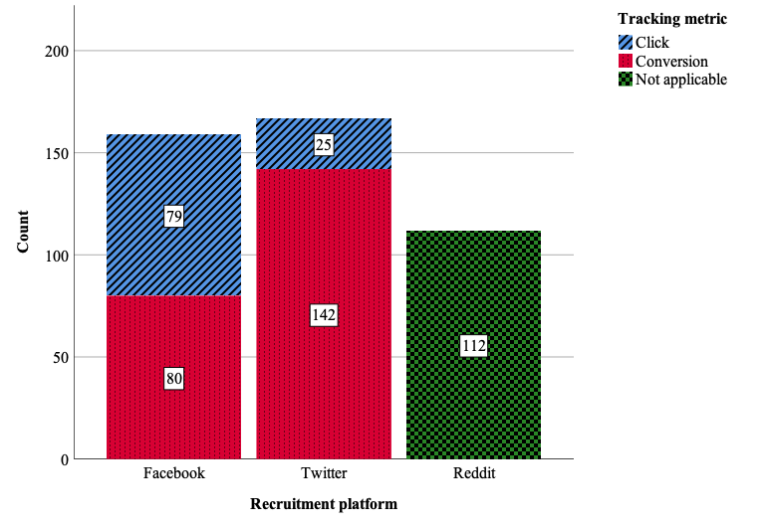
Respondent count by recruitment platform and tracking metric.

A total of 540 responses were received in the survey; however, 17 duplicate responses were identified during data cleaning, and an additional 88 respondents did not report having used a DTC-GT or did not report being aware of any third-party genetic interpretation companies and were subsequently excluded from the final sample (*N*=438). There were significant differences in the frequency of survey responses between the different experimental conditions (χ^2^_3_=84.2; *P*<.001). See Figure 3 for frequencies and Table 2 for pairwise comparisons. Nearly equal samples were collected from paid campaigns on Facebook (159/438, 36.3%) and Twitter (167/438, 38.1%). A significant but somewhat smaller sample of participants was recruited through the parallel unpaid campaign on Reddit (112/438, 25.6%). Of the participants recruited through paid campaigns (n=326), significantly more were recruited through the conversion-tracking campaigns (222/326, 68.1%) than through the click-tracking campaigns (104/326, 31.9%; *Z*=6.5; *P*<.001). The difference between conversion-based and click-based tracking metrics was much more pronounced on Twitter than on Facebook (*Z*=6.7, *P*<.001); correspondingly, of the 5 recruitment methodologies used, the Twitter-Conversion campaign recruited the greatest number of participants (142/438, 32.4%) and the Twitter-Click campaign recruited the fewest (25/438, 5.7%).

**Table 2:** Pairwise tests comparing the number of respondents recruited in paid conditions (Bonferroni-corrected threshold for statistical significance is alpha=.01).

Condition 1 (n)	Condition 2 (n)	*Z* score	*P* value
Facebook-Click (79)	Twitter-Click (25)	5.3	<.001
Facebook-Conversion (80)	Twitter-Conversion (142)	3.3	<.001
Facebook-Click (79)	Twitter-Conversion (142)	4.3	<.001
Facebook-Conversion (80)	Twitter-Click (25)	5.4	<.001
Twitter-Click (25)	Twitter-Conversion (142)	9.1	<.001
Facebook-Click (79)	Facebook-Conversion (80)	0.1	.94

### Cost-Effectiveness

Conversion-tracking campaigns on both Facebook and Twitter were more cost-effective at garnering survey respondents, averaging US $3.13 and US $1.76 per response, respectively. Click-based campaigns cost an average of US $3.16 and US $10.00 per response on the same platforms. There was a substantial difference in the cost of impressions between platforms in the click-based conditions, with Facebook charging US $1.91 per 1,000 impressions compared with US $9.90 on Twitter. There was, however, only a nominal difference in the cost of impressions among conversion-based conditions.

**Figure 4:**
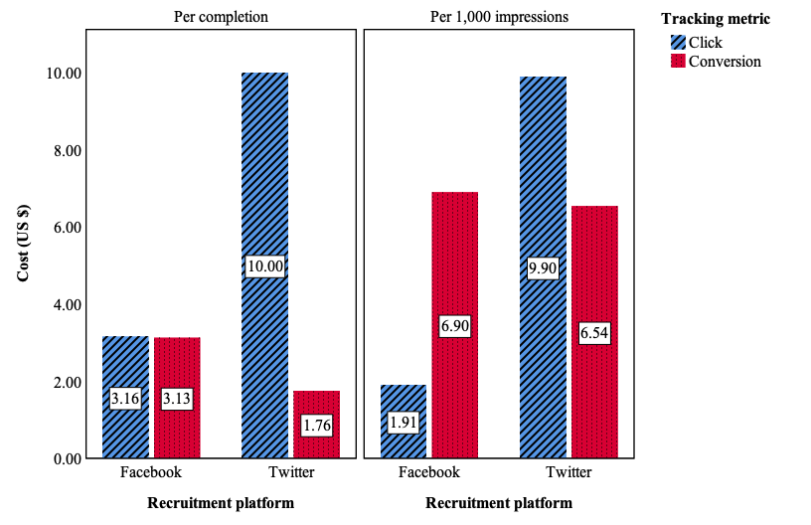
Cost-effectiveness measures by recruitment method.

Of the 4 paid advertising conditions, the Twitter-Conversion campaign was the most cost-effective in terms of generating survey responses, followed by the Facebook-Conversion, Facebook-Click, and Twitter-Click campaigns. The Facebook-Click campaign was the most cost-effective in generating broad audience exposure, followed by the Twitter-Conversion, Facebook-Conversion, and Twitter-Click campaigns (Figure 4).

### Demographic Comparisons Across Platforms and Tracking Methods

#### Differences Between Platforms

There were notable differences in the demographic characteristics of survey respondents recruited on each of the 3 platforms (Facebook, Twitter, and Reddit), particularly by age, gender, and race and ethnicity. There was no significant difference in the level of education reported by respondents recruited across different platforms (H_2=_ 4.61, *P*=.10).

Among those who reported their age (n=266), there were significant differences in the average age of respondents recruited on different platforms (*F*_2,263_=58.18; *P*<.001). The age distributions for respondents recruited on each platform are presented in Table 3. There was not a significant difference between the age of respondents recruited on Facebook (mean 49.13) and Twitter (mean 53.11); however, respondents recruited on Reddit were, on average, significantly younger than either of the other 2 groups (mean 34.23).

**Figure 5:**
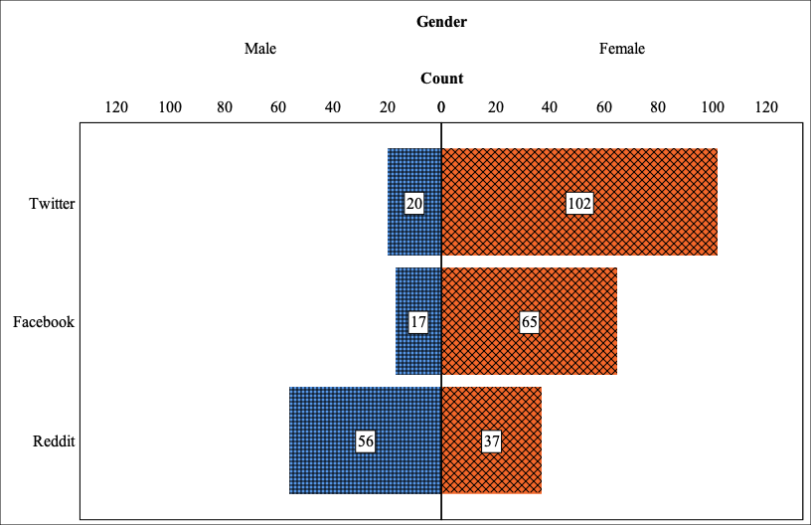
Distribution of respondent gender by recruitment platform.

There were significant differences in the ratio of female to male respondents between those who were recruited on different platforms (χ^2^_2_=53.0; *P*<.001). The gender distributions for each recruitment platform are presented in Figure 5. Female respondents made up the majority on both Facebook (65/82, 79%) and Twitter (102/122, 83.6%), but were in the minority among those recruited on Reddit (37/94, 39%).

Table 4 contains a complete reporting of respondent race and ethnicity by recruitment platform. The difference in the proportion of white to nonwhite respondents across platforms approached significance (χ^2^_2_=5.7; *P*=.06). The proportion of white respondents was higher on Twitter (105/121, 86.8%) than on Facebook (58/79, 73%) or Reddit (75/94, 80%).

**Table 3:** Respondent age by recruitment platform and tracking metric.

Category		Age (years), mean (SD)	*F* test (*df1*,*df2*)	*P* value
**Recruitment platform (n)**				
	Facebook (69)	49.13 (12.64)	58.18 (2,263)	<.001
	Twitter (110)	53.11 (12.95)	58.18 (2,263)	<.001
	Reddit (87)	34.23 (11.89)	58.18 (2,263)	<.001
	Overall (266)	45.90 (15.00)	—^a^	—
**Tracking metric (n)**				
	Click (50)	51.93 (12.67)	0.35 (1,177)	.56
	Conversion (129)	51.81 (13.08)	0.35 (1,177)	.56
	Overall (179)	51.58 (12.94)	—	—

^a^Not applicable.

**Table 4:** Respondent race and ethnicity by recruitment platform and tracking metric.

Category		Race and ethnicity					
		White/Caucasian	African American	Hispanic/Latino	Asian	Multiethnic	Other
**Recruitment platform**							
	Facebook (N=79)	58 (73)	2 (3)	5 (6)	1 (1)	8 (10)	5 (6)
	Twitter (N=121)	105 (86.8)	3 (2.5)	4 (3.3)	2 (1.7)	5 (4.1)	2 (1.7)
	Reddit (N=94)	75 (80)	2 (2)	1 (1)	5 (5)	10 (11)	1 (1)
	Overall (N=294)	238 (81.0)	7 (2.4)	10 (3.4)	8 (2.7)	23 (7.8)	8 (2.7)
**Tracking metric**							
	Click (N=57)	44 (77)	2 (4)	2 (4)	0 (0)	6 (11)	3 (5)
	Conversion (N=143)	119 (83.2)	3 (2.1)	7 (4.9)	3 (2.1)	7 (4.9)	4 (2.8)
	Overall (N=200)	163 (81.5)	5 (2.5)	9 (4.5)	3 (1.5)	13 (6.5)	7 (3.5)

^a^Respondents who selected more than 1 option for race and ethnicity.

**Figure 6:**
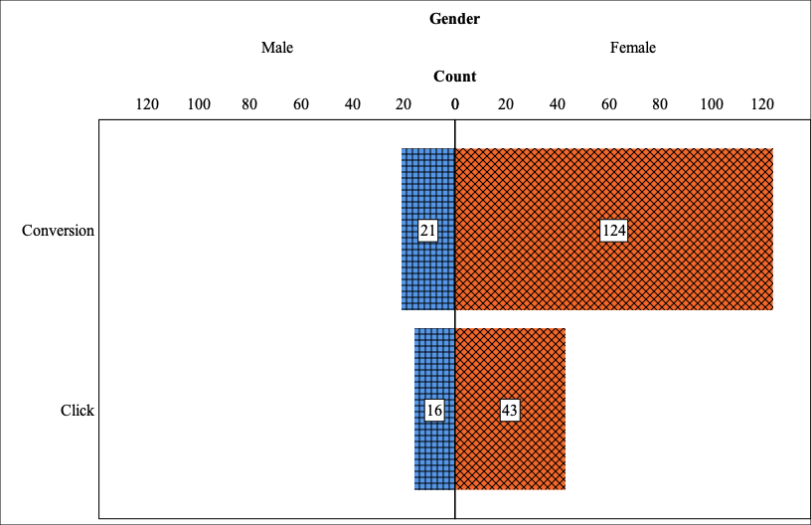
Distribution of respondent gender by tracking metric.

#### Differences Between Tracking Methods

The demographic characteristics of survey respondents recruited on paid advertising platforms (ie, Facebook and Twitter) using click-tracking were compared with those recruited using conversion-tracking. Respondents recruited from Reddit were excluded from this analysis, as no tracking method was used on this platform. The demographic differences between tracking methods were generally less substantial than those observed between platforms. The only significant difference noted was in the ratio of female to male respondents between those recruited using different tracking methods (χ^2^_1_=4.5; *P*=.03). Gender distributions for each tracking method are summarized in Figure 6. Female respondents made up the majority in both cases, but were more prevalent among those recruited using conversion-tracking (124/145, 85.5%), compared with those recruited using click-tracking (43/49, 73%).

There was no significant difference in the age distribution of respondents recruited using conversion-tracking compared with click-tracking (*F*_1,177_=0.35, *P*=.56) or in their level of education (H_1_=0.02, *P*=.88). Similarly, no significant differences were found between tracking methods in the proportion of white to nonwhite respondents recruited (χ^2^_1_=1.0, *P*=.32).

## Discussion

This study set out to test and evaluate the use of social media platforms as a recruitment tool for research on a hard-to-reach population. To do so, it directly compared paid and unpaid recruitment campaigns implemented on multiple social media platforms (Facebook, Twitter, and Reddit) and employed different advertising tracking metrics (click-based and conversion-based). Only a handful of studies have directly compared multiple methods of survey recruitment on social media; thus, this study represents a novel contribution to the development of Web-based survey methodology in general and recruitment approaches for hard-to-reach populations in particular.

Nearly identical sample sizes were obtained via paid Facebook (n=168) and Twitter (n=170) advertising, as well as the sample obtained via unpaid posting on Reddit (n=114). Although survey recruitment on social media for population health research has predominantly taken place on Facebook, this finding suggests that targeted advertising via other social media platforms may also be viable. Although the overall user base of Twitter is often noted to be substantially smaller than that of Facebook, and this was reflected in the reach of recruitment campaigns on that platform, this did not appear to constrain the effectiveness of Twitter as a platform for recruiting participants, with both Facebook and Twitter yielding comparable numbers of participants over both tracking metrics. Similarly, unpaid posting to community groups may also prove productive in achieving a broader sample.

The difference in the effectiveness of survey recruitment on Facebook and Twitter when considering both tracking metrics was found to be negligible; however, significant differences in the effectiveness of different tracking metrics across platforms were observed. Conversion-tracking campaigns recruited more than twice the number of respondents recruited by click-tracking campaigns, given the same budget. These results suggest that the use of different tracking metrics has important implications in determining the success of survey recruitment campaigns and warrants further investigation.

Overall, the use of conversion-tracking on Twitter was found to be the most cost-effective combination of tracking metric and platform conditions. The effectiveness of this approach may have been partially driven by organic growth (ie, individual users reposting recruitment materials from their own accounts). The number of survey responses garnered by this campaign exceeded the number of clicks detected on the advertisement, suggesting that this version of the recruitment materials was shared beyond the initial target audience for the advertising campaign. In an open-response question attached to the Reddit version of the questionnaire that asked respondents to identify the subreddit through which they had been recruited, 4 respondents indicated that recruitment materials had been forwarded to them by a friend or family member. Although these are the clearest indications for organic growth among the study conditions, it is possible that similar redistribution may have occurred in other cases as well.

This observation should serve as a reminder that all Web-based survey recruitment materials have the potential to be redistributed beyond the initial target audience, or otherwise *go viral*, unless steps are taken to prevent this. This potential may be useful in recruiting a larger sample or if recruiting entirely on social media platforms that do not allow targeted advertising. Here, researchers may wish to adopt from the existing literature on predictors of advertising message virality [20,21]. There is also significant cause for concern in contexts where nontarget audiences may be undesirable because of the scope or subject matter of the survey. Web-based surveys dealing with contentious topics have, in recent years, been redistributed in partisan discussion groups with the goal of sending a political message through the community’s collective response [22,23]. Surveys regarding health issues where significant public controversy exists might likewise be subject to purposive redistribution with the intent of affecting the results, even if initially targeted at a more limited audience. Future research should seek to identify the extent of organic sharing of survey recruitment materials and distinguish between the data collected from targeted and nontargeted respondents.

Demographic differences among study participants were observed between social media platforms and tracking methods. It should be noted that observed differences in the demographic makeup of samples apply only to the subset of participants in each who answered optional demographic questions. Reddit attracted a sample that was approximately 15 years younger on average than that recruited from either Facebook or Twitter. In addition, the sample recruited from Reddit included far more male respondents than that of either Facebook or Twitter. No significant difference between platforms was observed for either education levels or race and ethnicity.

In terms of tracking methods, conversion-tracking resulted in a sample that included more women than that recruited through click-tracking. No other demographic differences were observed. It is possible, given the higher number of female respondents observed among those recruited on Facebook and Twitter, that conversion-based targeting may have skewed the sample even further toward female respondents by iteratively targeting female users at a higher rate than male users.

The demographic breakdown of survey participants closely matches that of other surveys conducted with DTC testing consumers [24,25], reflecting early adopters of this technology who are primarily white and highly educated. Although this study appeared to represent more females than males, past surveys have shown gender variation across DTC companies themselves, which may reflect demographic differences in user base [25]. Similarly, although recruitment on Reddit, compared with Facebook and Twitter, resulted in a very different set of respondents, it is unlikely that any of the samples is more intrinsically representative of anything beyond the respective platform’s user base. As such, conducting recruitment on multiple platforms likely facilitated access to a more demographically diverse set of respondents, which yielded a final sample population that was more consistent with the past studies.

### Limitations

A major limitation for any study of modern Web-based advertising is the issue of algorithm dynamics or *the changes made by engineers to improve the commercial service and by consumers in using that service* [26]. Research findings on the effectiveness of specific software tools are intrinsically limited by the potential of such tools to evolve and change in unpredictable ways. The platform features used in this study to select an audience may not be viable in the near future, and Facebook or Twitter may update their advertisement targeting algorithm to select interested groups more effectively or more narrowly than researchers intend.

Limitations also arise from differences in the affordances of advertising platforms for defining a target audience as well as parameters for the display and timing of advertisements. Although the practical implications of these distinctions may be negligible, they do undermine the ability of researchers to directly compare the performance of advertising materials on different platforms or otherwise require researchers to isolate the most salient points of comparison: for example, in this study, differences in the way Facebook and Twitter handled bidding for advertising space meant that campaigns could either be restricted to equal budgets or to equivalent timeframes, with the former ultimately being deemed more relevant to the research questions at hand.

There were also substantial differences in the affordances of each platform for displaying advertising materials: the amount and size of displayed text, as well as the availability and size of graphics, are constrained both by the technological limitations of the platform (eg, Twitter’s character limit) as well as community norms and expectations. This study adjusted the advertising materials displayed in each condition to best take advantage of the affordances of that platform: for example, more extensive copy was displayed to Reddit users before clicking on the recruitment link than users on Facebook or Twitter. Although this allowed the experiment to conform more closely to the norms of each platform, and thus supported its ecological validity, it does introduce further limitations on the direct comparability of results across platforms.

When conducting survey research, numerous considerations can influence the desired sample size. Although unpaid recruitment in Web-based community groups may perform comparably to recruitment via paid advertisements on social media, it should be noted that the number of potential respondents reached through paid advertising is more readily scalable, given a sufficient budget. By contrast, the potential audience in Web-based communities is limited by the number of active users, which may be quite small for hard-to-reach populations of interest. Such communities typically frown on repeated posting, further limiting the audience that may be reached to those who are active in the period immediately following the initial post. The inherent limits on the scope of populations that can be reached through Web-based communities may, therefore, render unpaid Web-based recruitment less effective than paid advertising for achieving larger sample sizes.

In addition, paid advertising platforms allowed for audiences to be targeted based on location. In cases of organic sharing of those advertisements, as well as recruitment in community groups, no similar controls are available. Future research should compare survey data from targeted audiences against those reached through organic growth. Likewise, an assessment of how particular tracking metrics may lead to targeting of particular demographic or interest groups is necessary to fully understand the implications of using Web-based advertising for survey recruitment. As audience targeting and tracking algorithms continue to develop, longitudinal sampling of the same small population may be useful to evaluate whether algorithm dynamics have significant effects on Web-based survey recruitment.

These limitations only stress the need for additional comparative studies of survey recruitment through different Web-based advertising platforms and tracking metrics. By understanding and evaluating the results of Web-based distribution, researchers can be aware of the effectiveness and limitations of various targeting and tracking approaches. Likewise, by comparing the characteristics of respondents from multiple recruitment campaigns, it is possible to test the effectiveness of the different methods in reaching target populations. The results of this study suggest that conversion-tracking metrics support more cost-effective survey recruitment than conventional designation of audience parameters accompanied by click-based tracking. However, algorithmic targeting of advertisements also poses problems for the reliability and reproducibility of survey research as sampling mechanisms may change in unpredictable ways.

### Conclusions

The results of this study indicate that there are meaningful differences between different approaches to Web-based survey recruitment. Advertisements on social media are a pragmatic method for survey recruitment, particularly within hard-to-reach populations and are most effective when combined with conversion-based tracking metrics. Recruitment through Web-based community groups is an effective complementary approach for reaching such populations and may give access to a more diverse sample than advertising alone. These tools must be used with due intentionality and an awareness of limitations so as to avoid potential pitfalls. Future research is needed to fully understand the effect of organic sharing and algorithm dynamics on the constitution of Web-based samples.

## Appendix

Multimedia Appendix 1 Reddit Campaign Details.

## Abbreviations

DTC: direct-to-consumer
DTC-GT: direct-to-consumer genetic testing
IP: internet protocol
