# Correction: The Search for Consumers of Web-Based Raw DNA Interpretation Services: Using Social Media to Target Hard-to-Reach Populations

**DOI:** 10.2196/15735

**Published:** 2019-08-13

**Authors:** Tiernan J Cahill, Blake Wertz, Qiankun Zhong, Andrew Parlato, John Donegan, Rebecca Forman, Supriya Manot, Tianyi Wu, Yazhu Xu, James J Cummings, Tricia Norkunas Cunningham, Catharine Wang

**Affiliations:** 1 Division of Emerging Media Boston University College of Communication Boston, MA United States; 2 Department of Community Health Sciences Boston University School of Public Health Boston, MA United States

“The Search for Consumers of Web-Based Raw DNA Interpretation Services: Using Social Media to Target Hard-to-Reach Populations” (J Med Internet Res 2019;21(7):e12980) contained a typographical error in the Results section within the subsection “Demographic Comparisons Across Platforms and Tracking Methods” under the heading “Differences Between Tracking Methods”. The proportion of female respondents among those recruited using click-tracking was reported as “(43/49, 73%)” but should have been “(43/59, 73%)” to be consistent with Figure 6. This error has been corrected and the sentence now reads as follows:

Female respondents made up the majority in both cases, but were more prevalent among those recruited using conversion-tracking (124/145, 85.5%), compared with those recruited using click-tracking (43/59, 73%).

Additionally, incorrect sample sizes were noted in the second paragraph of the Discussion. The value for Facebook was listed as n=168 but should have been n=159; the value for Twitter was listed as n=170 but should have been n=167; and the value for Reddit was listed as n=114 but should have been n=112 to be consistent with values already reported in the Results section. The errors have been corrected and the sentence now reads as follows:

Nearly identical sample sizes were obtained via paid Facebook (n=159) and Twitter (n=167) advertising, as well as the sample obtained via unpaid posting on Reddit (n=112).

The corrections will appear in the online version of the paper on the JMIR website on August 13, 2019, together with the publication of this correction notice. Because this was made after submission to PubMed, PubMed Central, and other full-text repositories, the corrected article also has been resubmitted to those repositories.

