# Supplementary material for: The Search for Consumers of Web-Based Raw DNA Interpretation Services: Using Social Media to Target Hard-to-Reach Populations
Source: J Med Internet Res. 2019 Jul 30;21(7):e12980. doi: 10.2196/12980 (PMC6691676; doi:10.2196/12980)
Supplement: Multimedia Appendix 1 [file jmir_v21i7e12980_app1.docx]

**Multimedia Appendix A: Reddit Campaign Details**

Table 1. DTC-GT subreddits used in Reddit campaign.^a^

| Subreddit address | Subscribers | Respondents |
| --- | --- | --- |
|  | *N* | *n* |
|  |  |  |
| /r/23andMe | 1,707 | 16 |
| /r/23andmeShare | 90 | 2 |
| /r/bioinformatics | 10,550 | 2 |
| /r/DNA | 83 | 1 |
| /r/Geneology | 10,329 | 23 |
| /r/genetics | 11,978 | 8 |
| /r/genomics | 3,272 | 2 |
| /r/Nootropics | 73,016 | 33 |
| /r/promethease | 435 | 7 |
| /r/SampleSize | 33,074 | 2 |
| /r/SNPedia | 663 | 7 |

^a^ /r/Health subreddit dropped due to community guidelines that prohibit the posting of non-news materials.

Textbox 1. Body text of recruitment post for Reddit campaign.

| Have you used genetic testing (DNA) interpretive services? Tell us about your experience!  We are seeking individuals who have used a third-party service (such as Promethease or Livewello) to interpret raw DNA results from personal genetic testing (such as that offered by Ancestry.com or 23andMe) to complete a brief survey about their experiences. Participants will not be asked for any medical or personal health information. Upon completion of the survey, you can enter into a drawing for one of three $25 Amazon gift certificates.  Please follow this link to begin the survey.  This study is being conducted through [INSTITUTION]. If you have any questions about the study, please contact [AUTHOR]. |
| --- |
